# Intralesional and subcutaneous application of Viscum album L. (European mistletoe) extract in cervical carcinoma in situ: A CARE compliant case report: Erratum

**DOI:** 10.1097/MD.0000000000013950

**Published:** 2018-12-28

In the article, “Intralesional and subcutaneous application of Viscum album L. (European mistletoe) extract in cervical carcinoma in situ: A CARE compliant case report”,^[1]^ which appeared in Volume 97, Issue 48 of *Medicine*, the authors would like to add the following sentence to the Acknowledgements sections “We are thankful to the “Stiftung Integrative Medizin, Stuttgart, Germany” and the “Christophorus Stiftung, Stuttgart, Germany” for financial support.”
